# Polypharmacy and relapse of schizophrenia: are they related?

**DOI:** 10.1192/j.eurpsy.2022.1975

**Published:** 2022-09-01

**Authors:** R. Softic, S. Osmanovic, N. Becarevic

**Affiliations:** 1 University Clinical Center Tuzla, Of Psychiatry, Tuzla, Bosnia and Herzegovina; 2 Health Center Brčko, Of Psychiatry, Brčko, Bosnia and Herzegovina

**Keywords:** schizophrénia, Relapse, Polypharmacy

## Abstract

**Introduction:**

Polypharmacy can be the cause of deliberate discontinuation of medication and consequent relapse of schizophrenia.

**Objectives:**

To establish the one-year rate of relapse in the patients with schizophrenia with regard to monotherapy or polypharmacy.

**Methods:**

The sample of all hospitalized patients with schizophrenia in a five-year period was analyzed. Descriptive statistics were used.

**Results:**

Total of 87 participants (57 women), the median age was 43 years. Antipsychotic monotherapy was used in 31 (35.6%) of the participants. In one year period, 32 (36.8%) of all participants had a relapse. Prior to relapse, significantly more participants were treated with polypharmacy (p<0,05).

**Conclusions:**

Antipsychotic polypharmacy is related to a higher rate of relapse in patients with schizophrenia.

**Disclosure:**

No significant relationships.

